# Engineered platforms for mimicking cardiac development and drug screening

**DOI:** 10.1007/s00018-024-05231-1

**Published:** 2024-04-25

**Authors:** Madison Stiefbold, Haokang Zhang, Leo Q. Wan

**Affiliations:** 1 Department of Biomedical Engineering, Rensselaer Polytechnic Institute, Biotech 2147, 110 8t Street, Troy, NY 12180 USA; 2 Center for Biotechnology and Interdisciplinary Studies, Rensselaer Polytechnic Institute, Troy, NY 12180 USA; 3 Department of Biological Sciences, Rensselaer Polytechnic Institute, Troy, NY 12180 USA; 4 Center for Modeling, Simulation, and Imaging in Medicine, Rensselaer Polytechnic Institute, Troy, NY 12180 USA

**Keywords:** Cardiac development, Cardiac morphogenesis, Congenital heart diseases, In vitro platforms, Organ-on-chip, Embryo-on-chip, Cardiac tissue engineering

## Abstract

Congenital heart defects are associated with significant health challenges, demanding a deep understanding of the underlying biological mechanisms and, thus, better devices or platforms that can recapitulate human cardiac development. The discovery of human pluripotent stem cells has substantially reduced the dependence on animal models. Recent advances in stem cell biology, genetic editing, omics, microfluidics, and sensor technologies have further enabled remarkable progress in the development of in vitro platforms with increased fidelity and efficiency. In this review, we provide an overview of advancements in in vitro cardiac development platforms, with a particular focus on technological innovation. We categorize these platforms into four areas: two-dimensional solid substrate cultures, engineered substrate architectures that enhance cellular functions, cardiac organoids, and embryos/explants-on-chip models. We conclude by addressing current limitations and presenting future perspectives.

## Introduction

Disturbance of cardiac development during embryonic and fetal stages can lead to congenital heart defects (CHD) with severe health effects, including death. About 1% of births in the United States have a CHD, and 0.2% have what is termed a critical congenital heart defect (CCHD) [1, 2]. CHDs can lead to severe neurodevelopmental issues and other disorders [1], and children with CCHDs have a one-year survival rate of less than 80% [3]. A wide range of factors are suspected to play a role in embryonic and fetal cardiac disease, including genetic mutations, maternal diseases such as gestational diabetes, drugs such as thalidomide, and other environmental factors [4]. However, the exact mechanisms underlying embryonic and fetal cardiac diseases remain elusive, partially due to a lack of model systems for assessing the effects of potential disease-causing factors on cardiac development.

To establish cardiac model systems for practical drug screening, the fidelity in reproduction of the biology of human cardiac development is critical. In heart development, cardiac progenitor cells from the mesoderm germ layer form the first and second heart fields. The first heart field begins by forming a structure termed the cardiac crescent, with progenitor cells from the second heart field located nearby. The cardiac crescent then fuses into a tubular structure with both an outflow tract and an inflow tract [5]. The initial cardiac tube consists of an outer layer of cardiomyocytes separated from an inner layer of endocardial cells separated by cardiac jelly. This contractile structure is approximately linear before it begins to bend to the right, in what is termed the “c-looping” stage, the first symmetry-breaking structure in the embryo. The initial heart tube continues to grow and loop through different stages, with the cardiac progenitors from the first and second heart fields beginning to differentiate in different cardiac lineages [5, 6]. Finally, the eventual four-chambered heart is formed, with the apex pointing to the left side. Ideally, a model system capable of truthfully recreating these critical morphogenesis events can be adopted for clinical assessments of risk factors in cardiac development in order to avoid or minimize embryo exposure to these factors in utero.

The current understanding of embryonic and fetal heart development primarily comes from studies using human tissue samples, animal models, in vitro models, and computational predictions. However, each type of model has inherent limitations. For instance, there are ethical restrictions on using human samples, with the key phases of embryonic cardiac development completely inaccessible [7]. Animal models have many similarities to human development, but they have critical differences in gene expression and overall cardiac structures, raising concerns about their suitability in clinical applications. Meanwhile, computational simulations generally emphasize the mechanical aspects of development and lack the complexity of in vivo biological processes.

Alternatively, in vitro cardiac models of development, specifically organ-on-chip (OOC) devices, are a rapidly developing technology that is poised to make significant breakthroughs in understanding cardiac development. These devices can use human-derived cells to reflect human biology, as well as be fabricated in a way that allows for high-throughput testing of congenital disease risk factors. Depending on the design of an in vitro device, it can model different features and stages of cardiac development. A wide range of cellular characteristics [8] can be evaluated to determine the applicability of a particular model to represent specific developmental features. These decisions have implications for the overall complexity, cost, throughput, and measurable data that can be gathered from the model.

Here, we describe in vitro cardiac development models that can be used to study cardiac development and for drug screening applications. These developmental models contain different structural complexity, from simple two-dimensional (2D) cell culture models, to engineered structure models with simple cell arrangements, to more complex organoid models and whole-embryo models.

### 2D cell culture

The simplest format for cardiac developmental models is the 2D culture of single cells or cell monolayers with cells relevant to cardiac development and physiology. Depending on the scope of the study, these models allow for snapshots of key developmental phases or monitoring of the overall developmental process from stem cells to cardiomyocytes, which may involve advanced sensors created via nano/microfabrication technologies. The important model components, such as cells (isolated from animal models or through stem cell differentiation) and culture conditions (static or microfluidic environment), are largely determined by the specific study focus.

The cellular component can be sourced from both humans and animals. However, due to ethical concerns, animal primary cells have traditionally been sourced at specific developmental ages. Cells from animal developmental models allow consistent characterization of the cell differentiation stages. For example, avian embryonic cell culture is a well-established model for studying heart development [9], while isolating cells from chicken or quail embryos at different stages enables studies of cardiac development at specific phases. Despite the similarities across vertebrate species, the key distinctions between avian and human development inevitably compromise its recapitulation fidelity in modeling human embryonic development [10]. An alternative would be using mammalian cells, such as murine or porcine cells, given their more relevant developmental process [11]. Human-derived cells are an attractive solution, as the correct markers for cardiac development are expected to appear under the right culture conditions. Immature cardiac markers can be expressed after differentiating human embryonic stem cells (ESCs) or induced pluripotent stem cells (iPSCs). Multiple differentiation routes have been stably established [12, 13] and used to model a variety of CHDs [14], as previously discussed elsewhere. These differentiated cells have been evaluated with a variety of methods to assess the accuracy of their biophysical [8] and genetic [15, 16] characteristics. More recent works have further advanced the accuracy in the recapitulation of in vivo differentiation by these in vitro cell culture models.

**Fig. 1:**
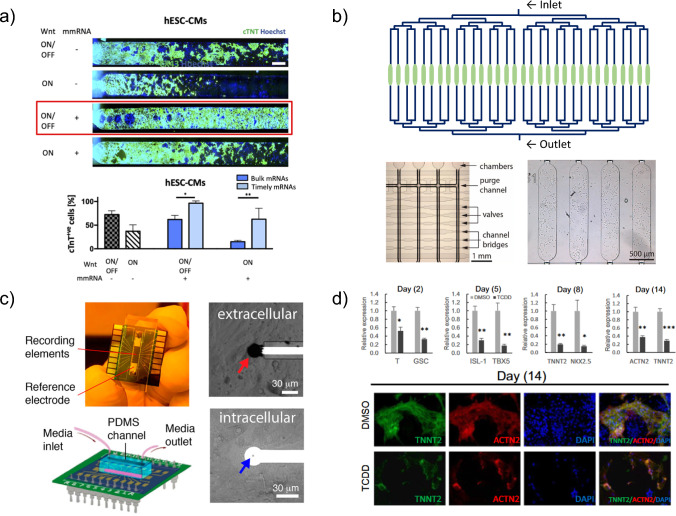
**a** Top, immunofluorescence of hESC-CMs cultured in a microfluidic device after undergoing differentiation with transient Wnt pathway activation (ON/OFF) or with continuous activation (ON), in conjunction with (+) and without (−) mmRNA transfection delivered in two phases. Cardiac marker cTNT (green), nuclei marker Hoechst (blue), scale bar 750 μm. Bottom, graph of percentage cells positive for cTNT marker with the four different Wnt and mmRNA combinations, with mmRNA delivered in either one (bulk) or two (timely) phases. Reproduced under terms of CC-BY license [26]. Copyright 2022, The Authors, and published by Frontiers in Bioengineering and Biotechnology. **b** Top, schematic of half the microfluidic chambers in the device described by the paper, with brightfield image of channels and valves (bottom left) and phase contrast image of cultured hESC cells (bottom right). Labeled microscopy images excerpted and rearranged from [29]. Reproduced under terms of CC BY 4.0 DEED license (https://creativecommons.org/licenses/by-nc-nd/3.0/commons.org/licenses/by/4.0/) [29]. Copyright 2021, The Authors, and published by Biomedical Microdevices. **c** Top left, image of chip device with electrodes, bottom left, schematic of PDMS cell culture area, top and bottom right, optical images of recording electrodes. Reproduced with permission from [34] (https://pubs.acs.org/doi/10.1021/acs.nanolett.0c00076). Copyright 2020, American Chemical Society, published by American Chemical Society (ACS). Further permission related to excerpted material should be directed to ACS [34]. **d** Bar plots, from let to right, compare the relative expression of mesoderm markers (T and GSC), cardiac markers (ISL-1, TBX5, and NkX2.5), and cardiomyocyte markers (ACTN2 and TNNT2) for hESCs undergoing cardiac differentiation and treated with either dimethylsulfoxide (DMSO) (light gray bars) or 2 nM 2,3,7,8‑Tetrachlorodibenzodioxin (TCDD) (dark gray bars). Immunofluorescence images show TNNT2 (green), ACTN2 (red), and nuclei (blue) in DMSO and TCDD-treated cells at day 14 of differentiation. Reproduced with permission from [42]. Copyright 2018, published by Elsevier B.V.

Advances in our understanding of cardiac differentiation and signaling factors have greatly improved the specificity and uniformity of cardiac lineage differentiations. For instance, the application of novel molecular techniques, such as single-cell RNA sequencing (scRNA-seq), has led to discoveries related to key cardiac markers and developmental pathways of different populations of cardiac progenitors [17–22], which enable the evaluation of the efficacy and specificity of in vitro stem cell differentiation to model the cell lineage of interest [23]. Closely controlled specificity of cardiac progenitor populations can navigate the cells to differentiate into specific cardiovascular progenitor populations [24] or more mature cardiac cell populations, such as cells with left ventricle-specific markers [25]. On the other hand, the differentiated cell populations can also be made with greater uniformity. For example, Contato et al. used synthetic modified messenger RNAs of transcription factors to differentiate both human ESCs and iPSCs within a microfluidic device, resulting in increased expression of cardiac markers over a more standard protocol (Fig. 1a) [26]. This technique may lead to decreased variability in gene expression levels in cells, which is advantageous for reproducibility in experiments. Utilizing the progress in manipulating cell differentiation, the in vitro cardiac models can achieve higher fidelity at the cell sourcing level.

In vitro models using differentiated cell cultures or primary animal cells have increasingly utilized the current improvements in fabrication techniques, sensors, and analytical approaches, giving rise to high-throughput platforms or devices. Differentiated cells can be further controlled through geometrically defined culture conditions. A common method is to use micropatterning techniques to create extracellular matrix (ECM) coated shapes on an otherwise non-cell adhesive surface. These can control the orientation of sarcomeres formed by differentiating cardiomyocytes, enabling comparisons between potential influences on cardiac development [27]. Alternatively, microfabrication techniques can create defined areas for cell attachment and growth. These can enable the cultured differentiating cardiomyocytes to be studied as they are cultured from a 2D state into a 3D microtissue [28]. Both micropatterning and nanofabrication techniques can be adapted for high-throughput platforms.

The development of high-throughput microfluidic platforms is advantageous for improved cell culture models. For example, Vollertsen et al. demonstrated a device with small-volume chambers (30 nanoliters) to tightly control the microenvironment of stem cells during their differentiation into cardiac progenitors, enabling more precise control and decreased use of expensive growth factors and medium (Fig. 1b) [29]. High-throughput platforms enable parallel drug screening or serve as efficient cytotoxicity assays that are directly applicable to various studies and in industry.

Improvements in sensors and analytical approaches can facilitate better characterization of the key signatures from the cardiac developmental models in different physiological development and pathological conditions. Critical cardiomyocyte features include electrical activity, calcium signaling, and force generation associated with cell contractility. Electrical activity associated with contraction can be detected with microelectrode arrays (MEAs), first applied to cells differentiated into cardiomyocytes over 20 years ago [30]. Advances in fabrication technology have seen these adapted for OOC devices [31, 32], with recent novel configurations such as nano-branched MEAs [33] or sensors embedded within chip devices to allow for recordings of both multicellular behavior and single-cell electrophysiological properties [34], thus providing greater analysis and insights into calcium signaling (Fig. 1c). Advanced microscopy techniques also enable the detection of calcium transients, whose analysis has been recently enhanced with the incorporation of machine learning techniques [35, 36]. Calcium signaling is closely related to cardiac cell contractility, which is necessary for the contractile properties of the linear heart tube [5]. Advances in atomic force microscopy (AFM) techniques to detect cell contractility [37] could be combined with cardiac development models, as contractility begins early with the formation of the linear heart tube. In addition, recent progress has made it possible for simultaneous detection of multiple aspects of the functionality of stem cell-derived cardiomyocytes [38], providing more complete observation and characterization of the cardiomyocyte functionality during different stages of cardiac development.

Cardiac development models with these evaluation technologies can be used to both further elucidate elements of cardiac development and study pathophysiological conditions. For example, ESC differentiations were used to identify the role of the canonical Wnt pathway in the development of pacemaker cells [39], and iPSC-derived cardiomyocytes were used to study the role of *DAND5* in altering cardiac differentiation and proliferation [40]. In contrast, pathophysiological studies often examine the role of developmental pathways in the context of genetic mutations or chemical perturbations. Both the TAZ mutation that causes Barth syndrome [27] and the CHD-causing mutations in gene TBX5 [41] were modeled in iPSC-derived cardiomyocytes. Chemical perturbations were studied by researchers in the expression of genetic markers during key developmental phases using an ESC cardiomyocyte differentiation model treated with 2,3,7,8‑tetrachlorodibenzo‑p‑dioxin (TCDD) (Fig. 1d) [42], or the changes in gene expression to link chemicals to disruption of specific pathways involved in cardiac differentiation [43]. More links between chemicals, genetics, or other environmental factors and the disruption of cardiac progenitor differentiation will likely be discovered in the future.

### Engineered substrate architecture

Models that pair disruption of cellular cardiac developmental pathways with the three-dimensional structure of in vivo tissue enable the study of additional modes of impaired development. The three-dimensional (3D) culture emphasizes the recapitulation of the overall in vivo tissue structure in addition to both the mechanical and chemical properties of the native cardiac tissue. The specific tissue structure of interest is modeled with engineered substrates that provide 3D constraints and guidance to cells, leading to tissue-level morphologies, behaviors, and functions that are otherwise not accessible for regular planar culture conditions. These engineered substrates, often extended into microfluidics devices with actuation, can serve as cardiac disease models or screening platforms that are still relatively simple and structured yet more biomimetic and versatile than 2D models.

**Fig. 2:**
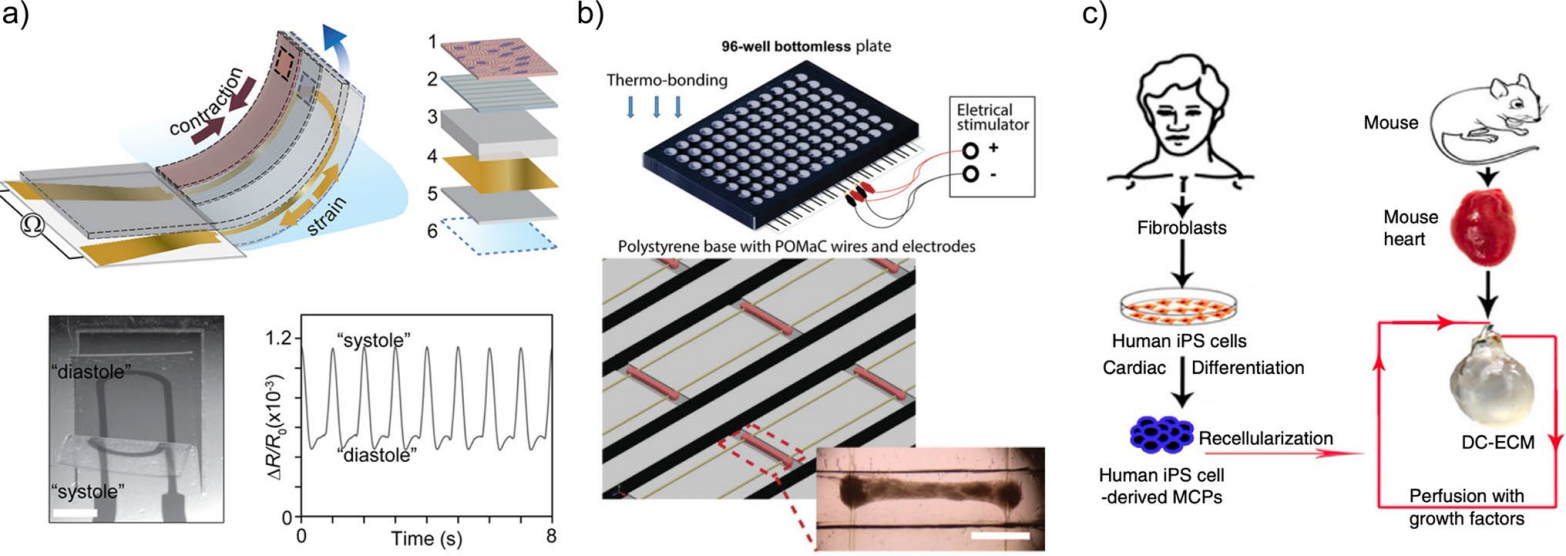
**a** Cardiac model with a PDMS cantilever and embedded strain gauge sensor. Top, a schematic of the layered structure of the cardiac cantilever with layers: (1) engineered cardiac muscle tissue, (2) tissue alignment layer, (3) PDMS, (4) Ti– Au thin-film strain gauge, (5) PDMS (6) poly(*N*-isopropylacrylamide) release layer. Bottom, an optical image of a deflecting cantilever and the corresponding results from the embedded strain gauge. Scale bar 1 mm. Reproduced from [49] with permission from the Royal Society of Chemistry. Copyright 2017, published by Royal Society of Chemistry. **b** Top, schematic of the Biowire 96-well plate embedded with electrical stimulators. Bottom, close-up of parallel wires and example cardiac tissue in device. Scale bar 1 mm. Adapted with permission from [52]. Copyright 2019 WILEY–VCH Verlag GmbH & Co. KGaA, Weinheim, published by John Wiley and Sons. **c** Schematic of repopulating a decellularized mouse heart with human iPSCs. Reproduced with permission from [62]. Copyright 2013, Springer Nature Limited, published by Springer Nature

A common technique for generating engineered substrates to recapitulate cardiac tissues in vitro is through fabricating polydimethylsiloxane (PDMS) into specific geometries for cell seeding. PDMS elastomer is widely used for organ-on-chip applications [44] given its advantageous characteristics such as high biocompatibility and simple fabrication via soft lithography. The surface of PDMS can be coated with ECM components such as fibronectin or adhesion RGD ligands to facilitate cell attachment, thereby promoting the morphogenesis of cell layers according to the surface features of the substrate [44–46]. For modeling cardiac tissues specifically, the tunable stiffness and elasticity of PDMS, as well as its resilience for repeated mechanical loading, are particularly important [46]. These characteristics are key for in vitro studies of cardiac muscle structures at this level, as these models often demonstrate repeated contraction-driven deformation or require stimulation of cyclic strain (Fig. 2a) [47–49]. As a typical PDMS-based design, muscular thin films (MTFs) are a common method to evaluate cardiomyocytes in vitro. Shim et al. [48] patterned ventricular myocytes onto fibronectin-coated PDMS strips to create MTFs, with one end of the cantilever beam anchored and the other end free-floating. Upon contraction, the cell layer bends the MTF up into a curve, and the contraction force can be calculated based on the curvature radius and the elastic modulus. Similarly, Wang et al. calculated the contraction stress from the shrinkage of the top projection length of the MTF caused by the upward curving [27]. Alternatively, Lind et al. embedded a flexible thin-film strain gauge within the PDMS layer of the MTFs and thus provided a continuous and more accurate readout of the contraction force with higher throughput [49]. These MTF devices can provide information about cardiomyocyte contractile properties at different stages of development, but they are limited in which features of development they can recapitulate. Instead of directly culturing cells on plain MTFs, pre-patterning the PDMS film substrates showed better manipulation and guiding of cell alignment. Sun et al. printed line micropatterns of various widths and spacing onto the MTFs prior to cell seeding. At optimal geometric parameters, the C2C12 myoblasts uniformly aligned in parallel along the patterned lines, which sufficiently guided and synchronized the contractile force direction upon stimulation, resulting in improved accuracy in force measurement and sample homogeneity [50].

More broadly, PDMS-based devices for modeling cardiac development suffer from multiple key characteristics related to its synthetic nature. The elastic modulus of PDMS is much higher than the native ECM environment in the developing heart, and such distinction in substrate mechanical properties is known to affect cell phenotype and behavior [46]. The synthetic composition of PDMS is also resistant to cell remodeling and infiltration. This limits the cell culture to monolayers, thereby causing difficulties in recapitulating the native cardiac tissues where cells arrange into multilayered or bundled structures inside the ECM.

Increased similarity to in vivo mechanical properties, tissue architecture, and potential for cellular remodeling can be achieved with hydrogels. With the emphasis on cell–matrix interaction, 3D devices with molded or casted hydrogels provide more biomimetic environmental cues. Variations in hydrogel composition, substrate stiffness, and chemical gradients can be optimized for modeling specific cardiac structures and disease conditions. For example, Zhao et al. [51] mixed a collagen hydrogel with atrial and ventricular cardiomyocytes and cast the mixture into a polystyrene chip with strip-shaped geometry, encapsulating polymer wires at both ends of the chip. After polymerization, the cellularized hydrogel strip formed compact cardiac tissues with separate regions of atrial and ventricular cardiomyocytes. These tissues contract under electrical pacing, applying detectable force to the wires. Their other work [52] further integrated the array of collagen strips, polymer wires, and electric pacing circuits into a bottomless 96-well plate-based chip (Fig. 2b), which significantly improved the throughput of the system as a drug testing/screening platform. This platform enables the drug screening platform to evaluate force generation, calcium transients, and gene expression in atrial versus ventricular tissue in a single device. However, devices with casted hydrogel such as this one only replicate a simple, isolated multicellular cardiac structure. PDMS and casted hydrogels often require the isolation of individual structures and simplification of designs for in vitro modeling in order to accommodate the technical challenges and limitations in fabrication.

Similar to PDMS devices, 3D-printed hydrogels can be formed into more complex biomimetic structures that model cardiac tissues. These 3D bioprinting techniques involve different strategies, including direct extrusion of cell-laden hydrogels [53], the extrusion-based method freeform reversible embedding of suspended hydrogels (FRESH) [54], and optical printing strategies [55]. These techniques can be combined with advances in creating more biomimetic bioink materials, resulting in an improved recapitulation of in vivo structural, mechanical, and chemical cues.

The most common 3D bioprinting processes are extrusion methods, which deposit the bioink layer by layer and allow for material crosslinking for 3D structures. However, common extrusion methods have difficulties in creating large structures because of the slow polymerization speed and the low material strength to support multilayered structures, eventually leading to the print collapsing. To solve these issues, the extrusion methods were recently enhanced by the FRESH technique, allowing for increasing the printing scale without loss of print fidelity due to the structural instability of bioink. The FRESH technique involves embedding the printing process inside a thermo-reversible support bath, preventing the collapse of bioink structures [56]. This enabled the fabrication of a cellularized linear heart tube model with spontaneous beating [54]. However, the FRESH technique and other bioprinting techniques often utilize bioinks that do not recapitulate the composition of in vivo ECM. As another alternative, the optical bioprinting methods generally include polymerizing the material deposited with illumination, such as the digital light processing (DLP) technique that uses UV or visible light wavelength range and the multiphoton lithography (MPL) technique that uses a laser, which is typically capable of stably generating 3D structures with features smaller than 50 µm. For instance, researchers using the DLP method were able to fabricate a patient-specific 3D model scaffold of a 30-week-old fetal heart and subsequently cultured cells in the model [57].

To improve the composition of the bioinks, decellularized ECM (dECM) can be incorporated. Taken directly from the relevant tissues, dECM scaffolds preserve the complex composition of proteins, proteoglycans, glycosaminoglycans, and other components. After processing and breaking down the dECM microstructures into liquid form, these soluble materials can be used similarly to normal hydrogels and molded into cell culture substrates or injected into damaged organs to facilitate repair [58]. Inks with dECM alone lack appropriate mechanical properties for bioprinting, but recent work in utilizing dECM in combination with other hydrogel components showed this mixture can improve the mechanical and chemical properties of substrates [59]. However, the use of soluble dECM components does not necessarily fully recapitulate the native architecture of ECM protein filament alignments.

The biomimicry can be further enhanced by using dECM scaffolds instead of isolated dECM components. These scaffolds retain their ECM structure [58, 59] and can provide an alternative to address the need for geometrical and compositional complexity. The scaffolds have shown success in cardiac tissue regeneration and repair [58]. Unprocessed and intact dECM scaffolds, such as whole heart scaffolds, can maintain both sophisticated geometrical features and the native vasculature structure, and these scaffolds have been used directly for recellularization and production of functional tissues and organs for in vitro modeling or transplantation purposes [60, 61]. Although ethical limitations prevent the use of dECM scaffolds from human embryos, in vitro models can still use animal sources of dECM to build improved substrates for human cell culture models. For example, Lu et al. repopulated decellularized mouse hearts with human iPSC-derived multipotential cardiovascular progenitor cells and reported that the cells could proliferate and differentiate in situ into cardiomyocytes, smooth muscle cells and endothelial cells, eventually forming an engineered heart with spontaneous contractions (Fig. 2c) [62].

The in vitro cardiac platforms at this level, in general, target mature cardiac tissues or structures for modeling, focusing on the recapitulation of the specific morphology, mechanical properties, and functional tissue properties with isolated or stem cell-derived cardiomyocytes [63–65]. Owing to the simplicity of the platform structures and the clarity of readouts (i.e., contractile profile), engineered substrates are generally presented with higher integration into microfluidic devices, microsensors, and corresponding analysis workflow, demonstrating great potential to be utilized as high throughput screening platforms or disease models. On the other hand, each engineered substrate model generally mimics a specific developmental stage, so establishing cardiac models via such platforms remains challenging. The use of 3D bioprinted scaffolds is promising, given the capability of printing cells within ECM structures for differentiation and development, but the system throughput is currently limited by fabrication efficiency and sample size.

### Guided development with organoids

3D cardiac structures of the developing embryo can also be studied by guiding the development of ESCs or iPSCs into 3D structures reminiscent of an embryo. These stem cells can be used to form embryoid bodies that recapitulate all three germ layers or only cardiac lineage-specific cells. Similar to 2D monolayer models with stem cell-derived cardiac cells, the guided development models in 3D can also be used to study the effects of genetic mutations or chemical perturbations on normal cardiac development by manipulating the differentiation pathways.

Embryoid bodies formed by stem cells can model early cardiac development along with the relevant mechanical and biochemical cues from the surrounding developing embryo. Recent work by the Zernicka-Goetz group has demonstrated the capability of mouse ESC-derived embryoids to form a beating heart structure [66, 67], which recapitulated some of the important features of early heart tube formation, including the expression of cardiac markers, tissue-level beating, and morphogenesis into heart tube structures. Future work utilizing this platform may examine the effects of genetic mutations or teratogen exposure on the embryoid body development.

**Fig. 3:**
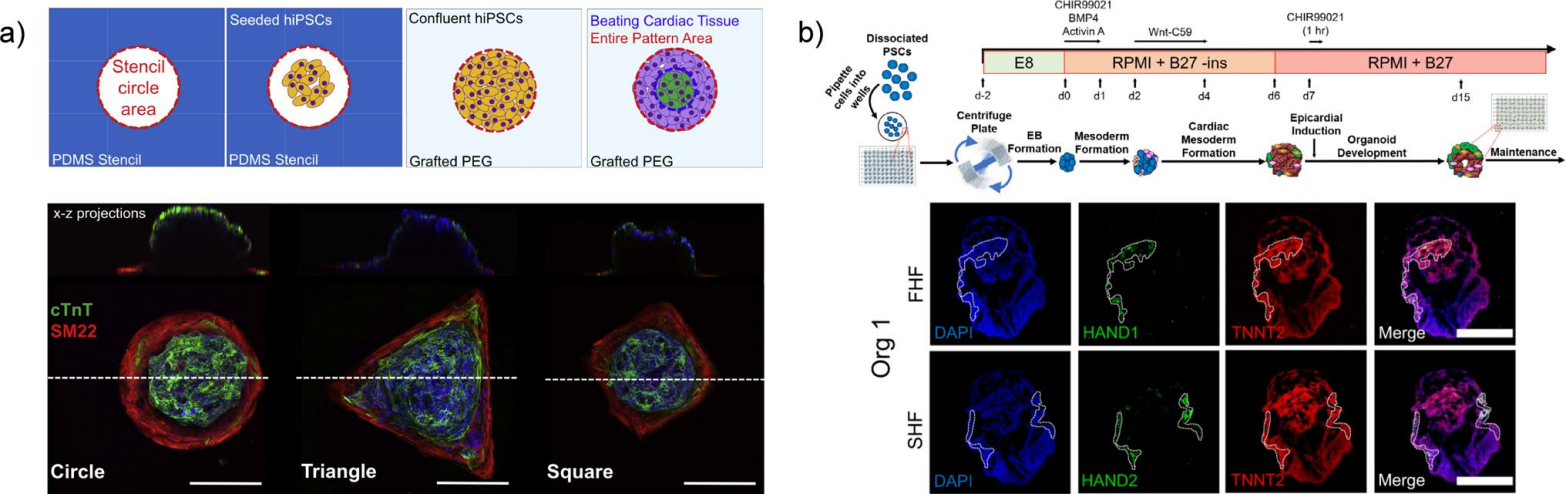
**a** Top, schematic of the formation of iPSC-derived cardiac organoids cultured on geometric shapes confined by a surrounding poly(ethylene glycol) (PEG) coated substrate. Bottom, immunofluorescent z-stack projection confocal images of cardiac marker cTnT (green), smooth muscle marker SM22 (green), and nuclei (blue). Scale bar is 200 μm. Adapted from [70] under license CC BY-NC-ND 4.0 DEED (https://creativecommons.org/licenses/by-nc-nd/4.0/). Copyright 2021, The Authors, published by Elsevier. **b** Top, schematic depicting protocol for forming iPSC-derived organoids by mimicking signaling of cardiac developmental stages using defined medium components. Bottom, cryosections of one iPSC-derived cardiac organoid on differentiation day 8 with labeling for nuclei (DAPI, blue), first heart field (FHF) marker HAND1 (green, top row), second heart field (SHF) marker HAND2 (green, bottom row), and cardiac marker TNNT2 (red). Adapted from [68], reproduced under license CC BY 4.0 DEED (http://creativecommons.org/licenses/by/4.0/). (Copyright 2021, published by Springer Nature Limited

More commonly, stem cells have been used to fabricate cardiac organoids by differentiating cell aggregates into cardiac-specific cell types and structures, which have then been used as in vitro cardiac development models or drug screening platforms. The cardiac organoids are made biologically similar to the in vivo cardiac environment, mainly by adding key cell types or other signaling factors to stem cell-derived cardiac cells (Fig. 3). The addition of additional cell types and other differentiation pathways has previously been accomplished with complex culture conditions and undefined cell culture components [68]. Progress in determining the key signaling factors for these models has shown promise in reducing complexity and cost. Recent work by Lewis-Israeli et al. demonstrates a method that uses fully defined cell culture media with only four signaling factors added at specific time points (Fig. 3b) [68]. This relative simplicity will make higher throughput screening more feasible by reducing the time and costs required for fabricating organoid models that model the entire developing heart. Alternatively, organoids can now be fabricated that recapitulate specific cell populations, such as cells with similar markers to those in the left ventricular wall [69]. These organoids will allow for more focused studies on pathophysiological states that affect specific areas of the developing heart. Cell populations have also been shown to be controlled by culturing organoids on micropatterned islands. This technique can create arrays of cardiac organoids with cell differentiations and contractile activity dependent on micropattern geometry (Fig. 3a) [70]. Combining these micropatterning techniques, specificity in cell populations, and simpler culture conditions will enable more controlled models with a greater amount of simultaneous organoid culture.

Recent organoid models have also incorporated embryonic tissues with progenitor cardiac tissue to study the interplay between the developing heart tube and other embryonic structures. For example, foregut development is thought to affect cardiac development and has been incorporated into developmental organoid models. Recent work has demonstrated the inclusion of early foregut development in cardiac organoids, with these organoids then recapitulating additional characteristics of cardiac development [71, 72]. Cardiac development has also been improved with the formation of multi-lineage gastruloids that recapitulate both cardiac and neural development [73], or multi-lineage organoids that include both pro-epicardium and liver bud that lead to epicardium and myocardium-like tissue layers [74]. These combinations of modeling cardiac development with other types of developing tissues appear to improve the phenotype of the cardiac lineage cells in the organoids. Non-cardiac cell signaling can also be introduced to cardiac organoids by engineering organ chips that contain multiple types of organoids [75]. Further work on incorporating non-cardiac cell lineage cells and structures with cardiac organoids will produce models that more closely replicate the different feedback systems that occur during development, making more accurate models for studying physiology and performing drug screening.

The potential for these cardiac organoids to be used for high-throughput drug screening depends on generating large numbers of reproducible organoids on a platform that enables efficient analysis of large numbers of samples. High throughput methods such as culturing organoids in well plates greatly increase the number of samples that can be processed in parallel [76]. Novel device designs that reduce required handling, such as perfused devices previously developed for 3D cell constructs [77], can be adapted for organoid culture. The large number of samples using these methods requires the use of advances made in the ability to image these samples. Improved optical clearing methods [78] will increase the data that can be captured through microscopical analysis. Demonstrations of the combination of optical clearing methods with microfluidic technology have shown that these can be used to increase the processing of samples in parallel [79]. Images of these cleared images can be analyzed with novel processing methods that can better analyze the tissue structures within organoids [80] and identify subpopulations of cells [81].

Cardiac organoids have already demonstrated their potential for applications in studying physiology, pathophysiology, and drug screening. For example, the roles of WNT-BMP and HAND1 in the formation of cardiac chambers were examined [82]. Pathophysiologic states replicated in organoids appear similar to phenotypes observed in vivo, such as gestational diabetes [68, 83] and genetic mutations (*e.g.*, a mutated Nkx2.5) [72]. Drug screening using cardiac organoids has been demonstrated by evaluating drugs with known side effects in these model systems. For example, Hoang et al. showed that drugs characterized by the FDA to have a low teratogenic risk did not have noticeable effects on the differentiation of cells in their organoid model, but those characterized as having a high teratogenic risk did result in defects in organoid development [70]. More in-depth evaluation of organoids treated with known teratogenic drugs will be needed to demonstrate that organoid models fully recapitulate the effects of these drugs during in vivo development.

### Natural development on a chip

**Fig. 4:**
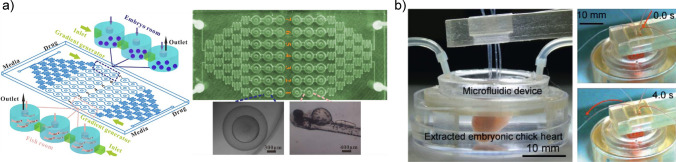
**a** Left, diagram of zebrafish assay chip with two independent areas for embryos and larva, each with three tanks for seven concentrations (C1-C7) derived from gradient generator. Right, photo and micrographs of the device with an embryo and a larvae fish. Adapted from [86] under CC BY 4.0 DEED (https://creativecommons.org/licenses/by/4.0/). Copyright 2014, Li et al., published by PLoS ONE. **b** Image of extracted embryonic chick heart in a microfluidic device with connected fluid flow between blood vessels and artificial tubes. Reproduced from [88] under CC BY-NC-ND 3.0 DEED licenses (https://creativecommons.org/licenses/by-nc-nd/3.0/). Copyright 2013, The Authors, published by Elsevier B.V.

To study cardiac development in vitro at the highest biological fidelity, researchers have taken advantage of natural development itself, integrating embryogenesis and cardiogenesis inside OOC devices (Fig. 4). These platforms, namely whole embryo chips [84–87] and organ explant chips [88, 89], enable direct investigation of cardiac development processes with minimal interference from the poor structural recapitulation of engineered tissues, but with compromises in technical challenges.

The common structure of whole embryo chips involves confining and culturing embryos inside an OOC device, allowing for batch culturing, drug/chemical supplementation, and live imaging. These devices provide various types of readouts to reflect embryo viability, development, environmental toxicity, and other responses. The current whole embryo chips predominantly culture zebrafish embryos or larvae instead of mammalian embryos, given the advantages of cost-effectiveness, low maintenance, accessibility, and large available quantity of these organisms [90]. A well-studied vertebrate model, the morphogenic and developmental basis of zebrafish is highly conserved among species, and the toxicity effects on zebrafish embryos are comparable to mammals [91]. Zebrafish embryos are permeable to most drugs and peptides, while the body transparency is compatible with optical microscopy [84, 90, 91]. Previous work using zebrafish embryos was mostly performed in well plates, leading to the problem of insufficient culture media and drug replenishment over the course of the development [84, 85]. Additionally, live imaging throughput is greatly limited due to the free-floating state of the embryos and, thus, an unfixed focal plane [84]. To address these issues, embryo culture was integrated into perfused microfluidic chips to achieve a more versatile control and higher experiment throughput. As an example, Choudhury et al. [84] created an imaging-compatible microfluidic platform with fish tanks connected to a series of fluidic gradient-generating channels, enabling dynamic embryogenesis under drug gradients for dose-dependent studies, while the small tank dimensions facilitate live imaging by locking the embryos in the plane of view. With the device, they observed the early development of multiple tissues and organs, as well as physiological processes, including blood flow under the effects of drugs. Targeting a later development stage, Fuad et al. [87] established a microfluidic chip that traps zebrafish larvae in individual flow channels to expose the larvae to drug stimulations, followed by characterization of cardiovascular activity by analyzing live recordings of heartbeats. Similarly, Li et al. [86] combined embryo and larva culture onto the same chip, allowing for chemical gradient generation and high throughput live imaging (Fig. 4a).

Despite the mechanistic similarities and experimental convenience of the zebrafish model, the cardiac structure and development remain significantly different from that of mammals. Thus, the information provided, which may be sufficient for rapid drug screening, is still limited for in-depth cardiac development studies. Previous investigations in the field have explored embryo culture for species more closely related to humans, such as porcine [92] and murine [93] models. As much as these works provided important insight into cardiac developments, they suffer from the complexity of the required culture environment, the long culture period, and difficulties in acquiring live readouts. As a result, these whole embryo cultures have proven challenging to integrate into OOC devices, and the throughput for currently available culture systems of pig and mouse embryos is relatively low for screening purposes.

Explant cardiac chips might be a viable alternative. Combining surgically extracted live hearts with perfused microfluidic devices, this type of chip allows for dynamic culture, drug or electric stimulation, live monitoring in vitro, and enables the use of much larger organs with more sophisticated and relevant structures without the difficulties of growing *ex utero* embryos. However, due to the use of mature cardiac tissues or organs, explant chip models to date, in general, focus on large-scale cardiac functions, such as contraction and pacing, instead of the cardiac development process. For instance, Owaki et al. [88] cultured the explanted chicken hearts inside a microfluidic device by connecting the major arteries to a peristaltic pump, which facilitates media flow and replenishment inside the explant. Up to four hearts can be maintained in the device simultaneously, and live imaging confirmed that the chicken hearts resumed beating with a medium supplement (Fig. 4b). Alternatively, Miller et al. [89] generated porcine heart tissue slices, instead of the whole heart, and mounted them into a tissue culture system allowing for electrical stimulation and drug supplementation. The contraction profiles of the slices can be analyzed by recording the slice movement, while the capability to maintain multiple slices simultaneously enables the throughput needed for a screening platform.

The OOC models at this level mainly focus on the development and functions of the entire cardiac tissue or organ and rely on the natural development of the embryos in vitro or in vivo instead of structural recapitulation with engineering approaches, while the microfluidic devices serve as versatile interface facilitating experimental efficiency and throughput. Both the current whole embryo chips and explant chip can be applied as drug or chemical screening platforms, with the whole embryo chips offering access to the entire development process at higher throughput and the explant chips enabling the use of organs or tissues from more relevant species pivoting the functions of mature cardiac structures. Although challenging, future studies may aim to study mammalian embryonic development inside microfluidic devices with reasonable throughput, potentially by miniaturization of current support systems for in vitro embryo culture and integration of live readout and analysis mechanics.

## Discussion and future directions

Current work in embryonic cardiac development models attempts to replicate different aspects of the structure and function of embryonic cardiac tissue. Much work on the differentiation pathways of cardiac cell precursors is starting to yield detailed information on the different populations of cells that make up the developing cardiac tissue at different developmental stages. In addition, in vivo animal models and the limited in vivo human tissue studies have led to a framework for the structure of the developing heart and its expected functionality. However, current cardiac developmental models include only select aspects of the structure or function of the developing cardiac tissue, with limited analysis of different functional readouts.

The replicability of cardiac models of different aspects of development is linked to their complexity and throughput, both important factors for drug screening applications. Microfluidic devices for analyzing cell monolayers of differentiating cardiac precursor cells can be used for cell-level functional assays. More complex models, such as organoids, can be used to study the organization of differentiating precursor cells at a tissue-level scale. These 3D cell aggregate models have a lower throughput than microfluidic devices and an increased complexity to their cell culture conditions. The tissue-level organization of cells in organoids does not yet fully recapitulate the tissue structures formed in vivo, such as a beating linear heart tube with a lumen. In order to model the structure of embryonic cardiac tissue, either engineered tissue structures or samples from natural development must be used. Engineered tissue structures of cardiac development can replicate specific tissue structures, but they do not progress through the different structural stages of cardiac development due to their static substrate morphology [57]. Similarly, explanted organs on chips will accurately replicate the cardiac tissue structure from its animal source but will not continue progressing through further structural development. In contrast, whole embryo microfluidic chips can be used to model gross structural changes, albeit with the differences in structures between an animal and human model.

These limitations in modeling cardiac function, structures, and transitions between developmental stages affect the applicability of these models. Model systems must be chosen for the ability to analyze a particular feature of interest. For example, studies analyzing the effects of drugs on the differentiation pathways of cardiac precursors could utilize high-throughput microfluidic chips with cardiac precursor cells. Studies focused on whether the genetic mutation in a patient’s DNA causes beating irregularities during development would need a model that recapitulates the development of contractile tissue and uses human cells, such as an organoid model. Different types of cardiac models vary in their ability to model different stages of cardiac development.

In addition to differences in the structure, function, and stage of development modeled, other factors, including throughput, complexity, and cost, must be considered when selecting a model. These characteristics will determine if a particular model can be used to study cardiac development or utilized for drug screening. The models discussed in this review have been used to make discoveries in cardiac development and replicate the effects of drugs known to perturb cardiac development. However, these models are not yet routinely used for drug screening applications.

To improve cardiac development models for translational use, progress needs to be made in several key areas. First, greater knowledge of cardiac development is required. The broad outlines of the structural stages of development and the cell populations involved are known, but many key details are unknown. The use of phenotypically accurate cells will increase confidence in the results from screening drugs that may perturb differentiation and, thus, development. Second, there is limited knowledge of the interactions between the developing cardiac tissue and the surrounding embryo. There may be key biomechanical forces or long-range intercellular signaling that affect cardiac development. Third, improved cellular model fabrication techniques are needed. Limitations in current techniques to control the 3D patterning and organization of cell aggregates, or the inclusion in engineered tissue models of features necessary for large 3D models, including fluid flow and vasculature, will prevent the modeling of the structural changes the developing heart tube undergoes. Both signaling pathways and mechanical forces will need to be considered in models of the c-looping process [6] and other stages of development. Fourth, improved fabrication and analysis techniques of chip devices are needed to increase throughput to levels needed for robust drug screening. Progress has been made for microfluidic devices that contain hundreds of samples of cells cultured in monolayers, but throughput levels for 3D models such as engineered tissues and organoids do not yet match this. In addition, the samples on each of these chips must then be analyzed, requiring the inclusion of electrodes for electrophysiology analyses, transparent substrates for imaging, or other features. Progress in these areas can lead to improved embryonic cardiac development models suitable for drug screening applications and eventually realize their potential to greatly reduce mortality and morbidity by preventing CHDs.

## Data Availability

This paper does not have associated data.
